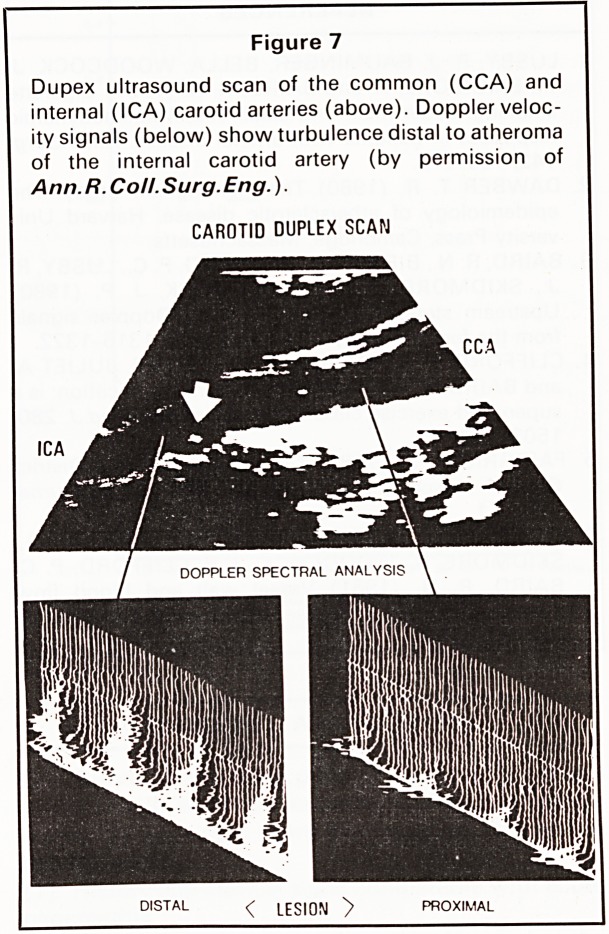# Surgery of the Circulation

**Published:** 1983

**Authors:** Roger N. Baird

**Affiliations:** Consultant Surgeon, Bristol Royal Infirmary and Cossham Hospital, Bristol


### Surgery of the Circulation

### (Long Fox Memorial Lecture, 11th November 1981, at the University of Bristol)

**Roger N. Baird, Ch.M., F.R.C.S.**
Consultant Surgeon, Bristol Royal Infirmary and Cossham Hospital, Bristol

### INTRODUCTION

I am honoured by the invitation of the University of Bristol and the Bristol Medico-Chirurgical Society to deliver the 1981 Long Fox Lecture.

Edward Long Fox, M.D., F.R.C.P. (1832–1902), in whose name this annual lecture is given, was physician at Bristol Royal Infirmary from 1857–1877. According to Rendle Short he was a splendid example of the best type of Victorian physician . . . an Infirmary man. Cecil Joll remembered his name as famous in the West Country when he was a schoolboy. Hey Groves described him as a great clinician and one whom by his research stimulated interest in medical science. These and other lecturers refer to the warmth of his personality and to his endearing qualities of simplicity, kindness and generosity.

Changes of awesome magnitude have taken place in medical and surgical practice in the 100 years which have elapsed since Long Fox's time. The operations for the surgical correction of atherosclerosis of lower limb arteries and carotid arteries which I shall discuss have been developed only in the past 30 years. The surgery of the circulation includes numerous other arterial and venous operations which cannot be mentioned tonight. The omissions clearly outweigh the inclusions as, for example, the burgeoning specialities of cardiac and microvascular surgery. These technical achievements have been made possible by advances in surgical, anaesthetic and nursing skills. There have been similar advances in arteriography, the main preoperative investigation. Most notable is the use of wire-guided, balloon-tipped catheters to recanalise and dilate narrowed arterial segments. Finally, ultrasonic investigation now plays an important role in the diagnosis of extra-cranial carotid artery disease and in the diagnosis of occlusive arterial disease of the lower limb.

### CARDIOVASCULAR RISK FACTORS

Most patients have a generalised arteriopathy with widespread disease affecting the coronary and cerebral arteries. Progression of disease may lead to myocardial infarction, stroke or restenosis of the treated arterial segment and can be largely prevented by correction of cardiovascular risk factors.

#### SMOKING

The most important risk factor is tobacco smoking and the effect on chronic limb arterial ischaemia of inhaling cigarette smoke is shown in Figure 1. Our studies with a Doppler probe applied to the skin over the posterior tibial artery have shown that pulsatile arterial flow-velocity is lost for 10–12 heartbeats following each inhalation.[1] Repeated exposure to these changes is probably the most important cause of permanent arterial damage in susceptible individuals.

**Figure 1**

Abolition of pulsatile blood flow at the ankle after inhaling smoke in a patient with foot ischaemia (by permission of ***Ann.R.Coll.Surg.Eng.***)

#### DIET

The relation of serum cholesterol to intermittent claudication is much weaker than for smoking. In the Framingham Study[2] those with the highest cholesterol levels were at greatest risk of claudication but otherwise no consistent gradient was noted. The hypothesis that reducing the animal fat and cholesterol content of the diet with restriction of calories to maintain an ideal weight remains unproved. Nevertheless the evidence is sufficiently convincing for many clinicians to give dietary advice to those at increased risk. As Henry V said to Falstaff:

> " Leave gourmandising: know the grave doth gape for thee thrice wider than for other men".

**EXERCISE**

Those with lower limb ischaemia tend to eschew physical exercise since they fear the onset of ischaemic pain. Some abstain from all physical activity. However, exercise training under supervision of a physiotherapist can substantially improve their walking distance: of 21 such patients recently studied, 19 achieved an 80% increase in their walking distance, two failed to improve with conservative measures and an arteriogram was done followed by arterial reconstruction.[3]

Patients with lower limb ischaemia are also examined for evidence of atherosclerosis elsewhere and particularly for the presence of an aortic aneurysm or a carotid bruit. Other correctable risk factors include hypertension and diabetes, and stress: those in demanding occupations are well advised to seek less stressful employment.

### LOWER LIMB ISCHAEMIA

The symptoms of chronic occlusive atherosclerosis of the lower limb arteries are intermittent claudication and ischaemic rest pain. The diagnosis is established by finding reduced or absent femoral, popliteal or pedal pulses on palpation. The severity and extent of vessel lumen encroachment are determined by x-ray contrast arteriography. Surgical reconstruction is done with tubes of vein or synthetic material or by endarterectomy. Radiologists have recently extended our therapeutic repertoire by skilfully manipulating balloon-tipped catheters to dilate narrowed arterial segments and recanalise arterial occlusions. These therapeutic procedures are highly effective in restoring many with intermittent claudication to their normal activities and in warming a severely ischaemic foot so that rest pain is relieved thus preventing a major amputation of the limb. Their long term success depends much on arresting the progression of atherosclerosis by correcting the cardiovascular risk factors referred to above.

### CONFIRMING THE DIAGNOSIS

**CLAUDICATION**

The history is always revealing. Most common is a cramp-like pain in the calf which accompanies occlusion of the superficial femoral artery in mid-thigh. On examination the femoral pulse is of good volume and distal pulses cannot be felt. If, on the other hand, the iliac arteries are primarily affected, there may be an exercise-induced ache or tired feeling in the thigh or buttock and the femoral pulse is weak or absent.

**NEUROGENIC CLAUDICATION**

Nerve entrapment from spinal stenosis or a prolapsed disc can cause exercise-induced pain which mimics vasculogenic claudication. Neurogenic claudication is suspected if there is a history of low back ache or if the symptoms include numbness, tingling or other paraesthesia of the legs. Exercise testing, pulse palpation and Doppler ankle pressure measurements can help to clarify whether the symptoms are of vascular or orthopaedic origin.

**OTHER CAUSES**

Osteo-arthritis, rheumatoid arthritis, gout and the anterior tibial compartment syndrome can give rise to symptoms suggestive of claudication.

Non-atheromatous causes of claudication include arterial embolism and the popliteal entrapment syndrome.

**PALPATING THE PULSE**

Examination of the arterial pulse is of cardinal importance. Often the findings are straightforward. For example, in severe iliac artery disease which is ripe for reconstruction, the femoral pulse is hard, cord-like and non-pulsatile. Sometimes the clinician is not sure:

> "I have more than once observed old and eminent practitioners make such different judgements of hard, full and weak and small pulses, that I was sure they did not call the same sensations by the same name." Heberden, 1768.

If the pulse is thought to be reduced, it can be re-examined immediately following exercise for diminuition of volume and loss of pulsatility. A harsh systolic murmur may be heard on auscultation of the femoral artery in the groin. Ultrasonic methods have been developed to quantitate reduced arterial pulsation in disease states.[4]

**ANKLE SYSTOLIC PRESSURE**

In normal subjects the systolic pressure at the ankle equals or exceeds the arm pressure. Reduction of the ankle pressure is a sensitive indicator of arterial disease. An inexpensive Doppler probe (Figure 2) will detect the arterial pulse even if it is impalpable. In well-collateralised arterial occlusions the resting ankle pressure may be nearly normal. Repeating the measurement following exercise to demonstrate a fall in pressure can help to confirm the diagnosis.

**Figure 2**

Doppler probe used to detect arterial flow-velocity in measuring ankle systolic pressure.

### TREATMENT

As mentioned earlier, a prime objective in the treatment of lower limb ischaemia is to stem the progression of atherosclerosis by reversing cardiovascular risk factors. Some patients have found relief from vasodilators or from garlic, onions, vitamin E and other remedies.

Two main lines of definitive treatment are available: surgical bypass, or recanalisation and dilation by the radiologists using a balloon-tipped catheter – percutaneous transluminal angioplasty.

*Arterial bypass* is most successful in aorto-iliac disease. Treatment of distal disease by grafts from the femoral artery to the popliteal or tibial vessels is less successful in the long term and these grafts are generally reserved for limb threatening ischaemia.

*Percutaneous transluminal angioplasty* (Figure 3) is currently enjoying widespread popularity for both proximal and distal disease. It is effective, and has the advantages of avoiding the discomfort of an operation and is virtually an outpatient procedure. However, some arterial lesions are not suitable for treatment, and the procedure carries risks of thrombosis and embolism. Preliminary results in our first 40–50 cases in Bristol are encouraging with short incomplete iliac artery stenoses dilating particularly well (Figure 4).

**Figure 3**

Diagram of balloon dilation of an arterial stenosis (by permission of ***The Medical Annual 1982/83***; John Wright · PSG).

**Figure 4**

Arteriograms before (left) and after (right) balloon dilation of a stenosed left common iliac artery (arrowed). A narrowed external iliac artery was also dilated (by permission of ***The Medical Annual 1982/83***: John Wright · PSG)

### CAROTID ARTERY STENOSIS

Atheroma at the origin of the internal carotid artery can result in reduced cerebral blood flow and discharge of debris which embolises to the eye or cerebral hemisphere (Figure 5). Eye symptoms range from a momentary curtain – like loss of vision (amaurosis fugax) – to complete blindness. Hemispheric transient ischaemic attacks (TIA's) typically present as a temporary motor or sensory loss affecting the arm, leg, face or speech. Auscultation of a localised murmur at the carotid bifurcation is the most reliable clinical indicator of carotid stenosis. Cerebral ischaemia causes anxiety because of the risk of a permanent stroke or blindness. When caused by carotid stenosis it is effectively treated by carotid endarterectomy which improves cerebral perfusion and eliminates a source of emboli. Good results were recently reported by Fairgrieve in the *Journal.*[5] With careful patient selection and an experienced surgical team the operative mortality and risk of perioperative neurological deficit are sufficiently small to encourage renewed confidence in this method of stroke prevention. For those TIA's without carotid

**Figure 5**

Mechanism of transient cerebral ischaemia. Discharge of fibrin/platelet and cholesterol debris from necrotic plaque of internal carotid artery.

**Figure 6**

Arteriogram of an atheromatous stenosis (arrowed) at the origin of the internal carotid artery.

disease, aspirin can provide relief of symptoms by its antiplatelet effect.

### INVESTIGATIONS

Carotid arteriography is the definitive preoperative investigation (Figure 6) and is proceeded to directly in straightforward, severe and worrying cases in whom operation is contemplated. In those with milder, atypical or infrequent symptoms and those in whom evidence of carotid disease is felt desirable prior to arteriography there are several useful non-invasive tests. In Bristol we have used Duplex ultrasound scanners employing both real-time and Doppler to show disease at the carotid bifurcation.[6] Turbulence caused by atheromatous encroachment of the vessel lumen is well detected using Doppler-shifted ultrasound (Figure 7).

**Figure 7**

Dupex ultrasound scan of the common (CCA) and internal (ICA) carotid arteries (above). Doppler velocity signals (below) show turbulence distal to atheroma of the internal carotid artery (by permission of ***Ann.R.Coll.Surg.Eng.***).

### CONCLUSIONS

Recent advances have provided a better understanding of the pathophysiology of occlusive atheroma of the lower limb and carotid arteries. The use of ultrasound measurements has complemented clinical evaluation and arteriography in providing a more precise assessment of the extent and effects of arterial disease as well as monitoring the results of treatment.

### ACKNOWLEDGEMENTS

For their advice, help and encouragement I thank many surgeons, anaesthetists, radiologists and medical physicists, especially the following: D. R. Bird, W. B. Campbell, P. C. Clifford, G. Cooper, E. Rhys Davies, W. D. Jeans, R. J. Lusby, J. H. Peacock, C. Prys-Roberts, R. Skidmore, P. N. T. Wells and J. P. Woodcock.

### REFERENCES

1. LUSBY, R. J. BAUMINGER, BELLA, WOODCOCK, J. P., SKIDMORE, R. and BAIRD, R. N. (1981) Cigarette smoking: acute main and small vessel haemodynamic responses in patients with arterial disease. *Am.J.Surg.* **142**, 169–173.
2. DAWBER T. R. (1980) The Framingham Study. The epidemiology of athersclerotic disease. Harvard University Press, Cambridge, Massachusetts.
3. BAIRD, R. N., BIRD, D. R., CLIFFORD, P. C., LUSBY, R. J., SKIDMORE, R. and WOODCOCK, J. P. (1980) Upstream stenosis. Its diagnosis by Doppler signals from the femoral artery. *Arch.Surg.* **115**, 1316–1322.
4. CLIFFORD, P. C., DAVIES, P. W., HAYNE, JULIET A. and BAIRD, R. N. (1980) Intermittent claudication: is a supervised exercise class worthwhile? *Brit.Med.J.* **280**, 1503–1505.
5. FAIRGRIEVE, J. (1981) Carotid surgery in a District General Hospital. *Bristol Medico-Chirurgical Journal* **96**, 8–11.
6. LUSBY, R. J., MACHLEDER, H. I., JEANS, W. D., SKIDMORE, R., WOODCOCK, J. P. CLIFFORD, P. C. BAIRD, R. N. (1981) Vessel wall and blood flow dynamics in arterial disease. *Phil.Trans.R.Soc.Lond. B* **294**, 231–239.

## Figures and Tables

**Figure 1 f1:**
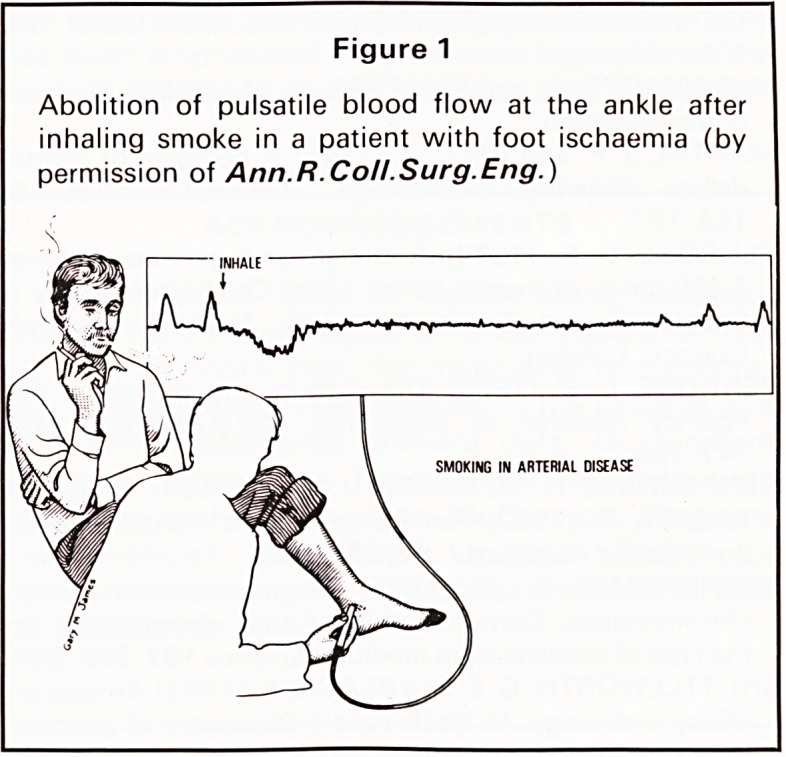


**Figure 2 f2:**
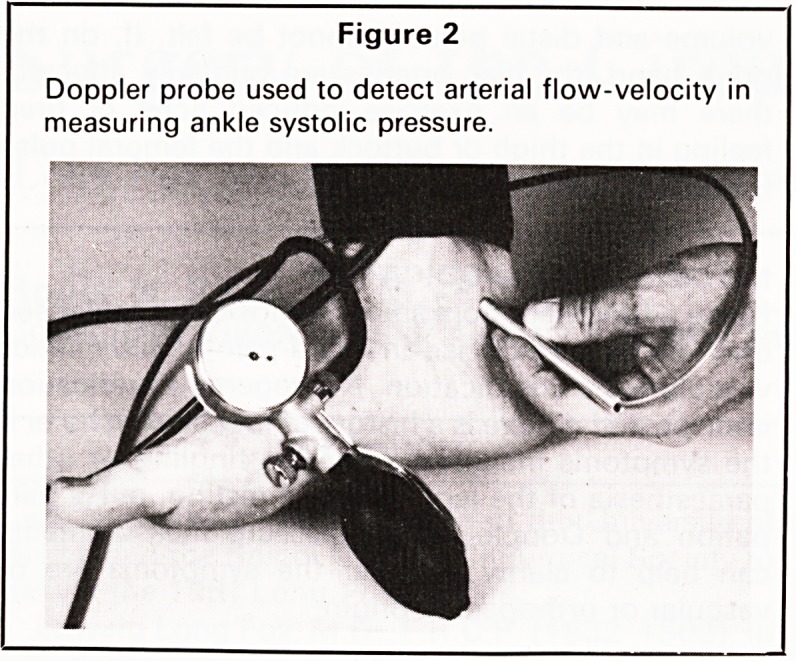


**Figure 3 f3:**
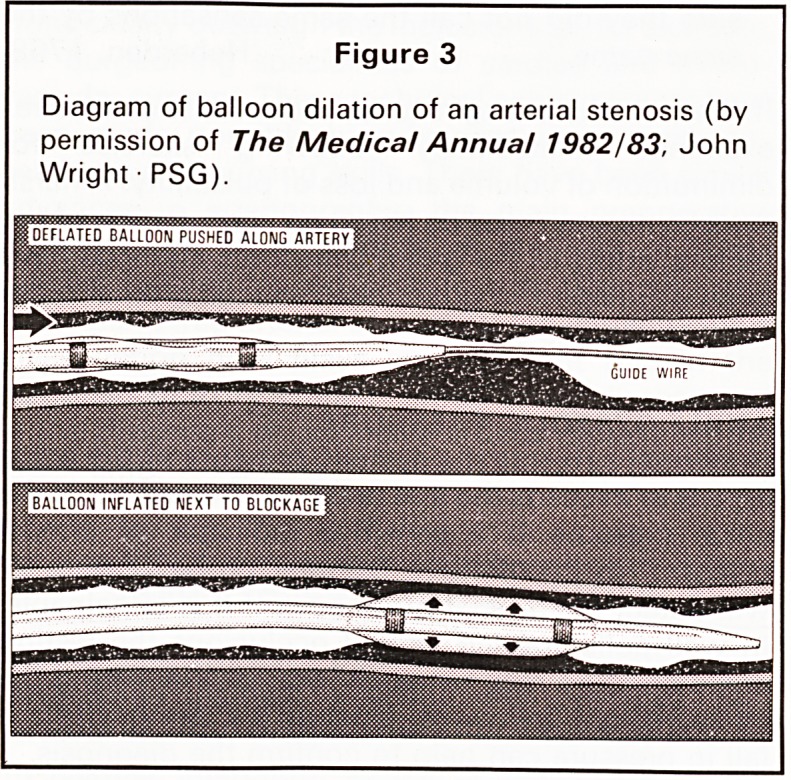


**Figure 4 f4:**
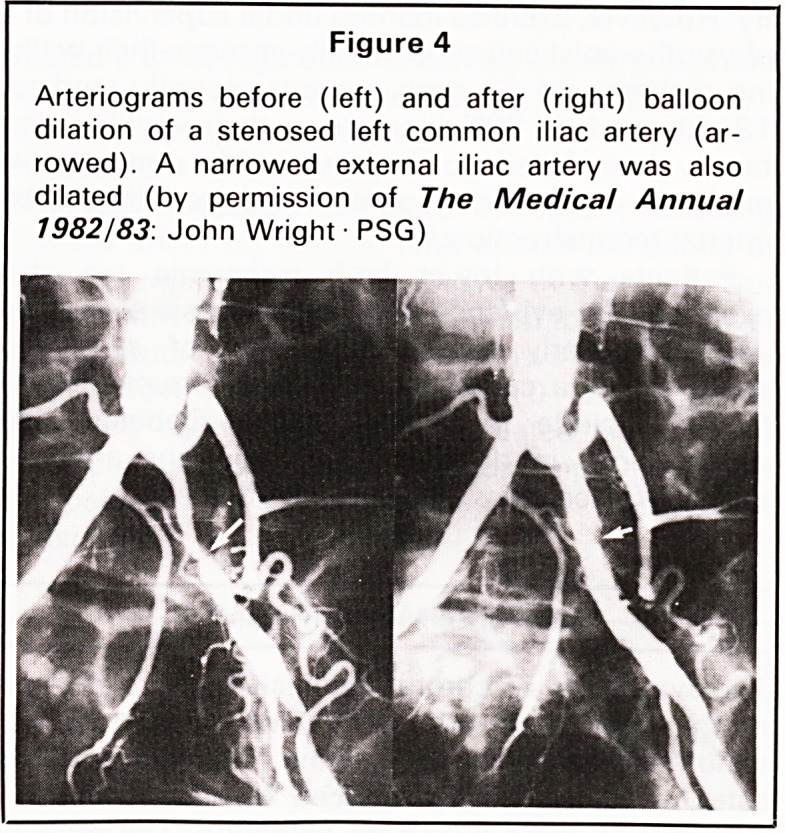


**Figure 5 f5:**
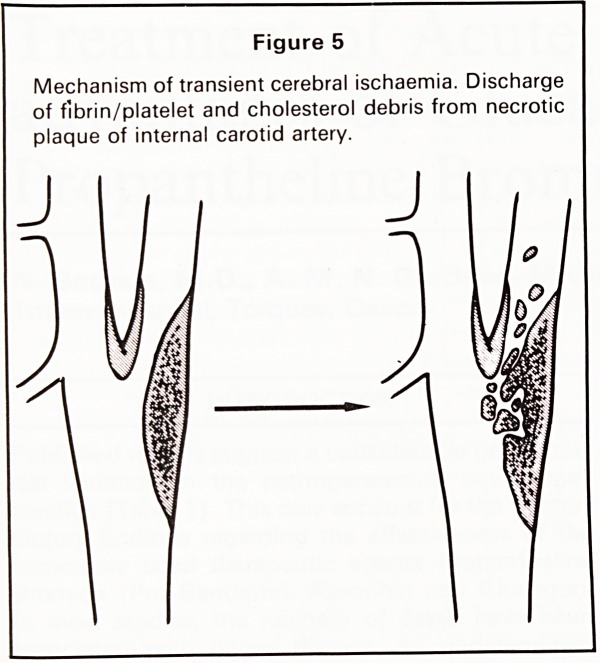


**Figure 6 f6:**
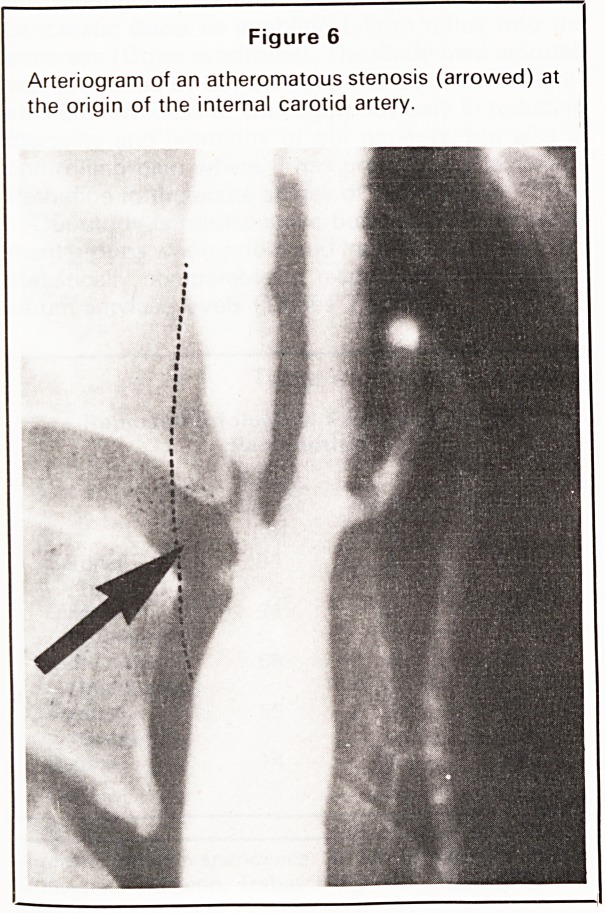


**Figure 7 f7:**